# The Differences between Luminal Microbiota and Mucosal Microbiota in Mice

**DOI:** 10.4014/jmb.1908.08037

**Published:** 2019-10-22

**Authors:** Minna Wu, Puze Li, Jianmin Li, Yunying An, Mingyong Wang, Genshen Zhong

**Affiliations:** 1 Xinxiang Key Laboratory of Pathogenic Biology, School of Basic Medical Sciences, Xinxiang Medical University, Xinxiang 453003, Henan, P.R. China; 2 Henan Key Laboratory of Immunology and Targeted Therapy, Henan Collaborative Innovation Center of Molecular Diagnosis and Laboratory Medicine, Xinxiang Medical University, Xinxiang 453003, Henan, P.R. China

**Keywords:** Luminal microbiota, mucosal microbiota, sequencing, duodenum, colon

## Abstract

The differences between luminal microbiota (LM) and mucosal microbiota (MAM) were little known, especially in duodenum. In this study, LM and MAM in colon and duodenum of mice were investigated through 16S rRNA high-throughput sequencing. The lowest bacterial diversity and evenness were observed in duodenal LM (D_LM), followed by duodenal MAM (D_MAM). Meanwhile, the bacterial diversity and evenness were obviously increased in D_MAM than these in D_LM, while no significant difference was observed between colonic MAM (C_MAM) and colonic LM (C_LM). PCoA analysis also showed that bacterial communities of LM and MAM in duodenum were completely separated, while these in colon overlapped partly. The ratio of Firmicutes to Bacteroidetes (F/B) in D_MAM was significantly higher than that in D_LM. *Lactobacillus* was largely enriched and was the characteristic bacteria in D_LM. The characteristic bacteria in D_MAM were *Turicibacter*, *Parasutterella*, *Marvinbryantia* and *Bifidobacterium*, while in C_LM they were *Ruminiclostridium*_6, *Ruminiclostridium*_9, *Ruminococcaceae*_UCG_007 and *Lachnospiraceae*_UCG_010, and in C_MAM they were *Lachnospiraceae*_NK4A136, *Mucispirillum*, *Alistipes*, *Ruminiclostridium* and *Odoribacter*. The networks showed that more interactions existed in colonic microbiota (24 nodes and 74 edges) than in duodenal microbiota (17 nodes and 29 edges). The 16S rDNA function prediction results indicated that bigger differences of function exist between LM and MAM in duodenum than these in colon. In conclusion, microbiota from intestinal luminal content and mucosa were different both in colon and in duodenum, and bacteria in colon interacted with each other much more closely than those in duodenum.

## Introduction

Trillions of microbes, referred to as microbiota, colonize the intestinal tract of animals and humans. The Metagenomics of Human Intestinal Tract (MetaHIT) project discovered that there are 1,000-1,150 kinds of bacteria in the human gastrointestinal tract, including *Bacteroides*, *Clostridium*, *Lactobacillus* and *Escherichia*. Most of these are anaerobic bacteria, with the number exceeding 10^14^, or about 10 times that of human cells [1]. Recently, many studies found that the gastrointestinal (GI) microbiome not only makes essential products and forms a barrier against pathogens, but also has multiple functions in immunity development, digestion, nutrition, and acute and chronic infections. In fact, microbes participate in the host’s physiological, pathological and pharmacological (toxicological) processes, forming an important part of the metabolic network [2-4]. The intestinal microbiota is an enormous and dynamic system; its imbalance is closely related to the occurrence and development of many diseases, such as gastrointestinal diseases (inflammatory bowel disease, colorectal inflammation/colorectal cancer), metabolic diseases (obesity and type 2 diabetes), respiratory diseases (asthma), cardiovascular and cerebrovascular diseases (stroke), neurological diseases (Parkinson’s disease, autism), etc. [5, 6].

The intestinal mucosa is a mechanical barrier composed of mucosal epithelial cells. Due to the existence of plicae, villi and microvilli, the area of the intestinal tract of humans and other mammals is very large. For example, the area of an adult gastrointestinal mucosa can reach 400 m^2^, while the surface area of the human body is 1.5-2.0 m^2^ [7]. Therefore, the intestinal mucosa is the largest gateway for material exchange between humans and the outside world.

The microorganisms that settle on intestinal mucosa are an important part of microbiota. Together with intestinal mucosa, mucin and secretory IgA, they are vital in blocking and delaying the translocation and infection of various pathogenic microorganisms [8, 9]. Several studies rhave so far evealed the differences between luminal microbiota (LM) and mucosal microbiota (MAM). The colonic LM and MAM in healthy individuals had distinct microbial ecosystems that differ significantly from each other in microbial diversity and composition [10]. Also, microbial compositions of colonic LM and MAM were different in patients with functional gastrointestinal disorders [11] or with rectal carcinoma [12] or with diarrhea-predominant irritable bowel syndrome [13]. However, little is known about the difference between LM and MAM in other intestinal locations except the colorectal region.

As the longest tube in the digestive tract, the microbial community compositions in different locations are also different. The microbial communities found in the small intestine and stomach of mice were different from those seen in the large intestine luminal content and fecal samples [14-16]. The dynamic distribution of porcine microbiota across gastrointestinal tract segments was also studied, and found that the dominant genera in the small intestine belonged to aerobe or facultative anaerobe categories, whereas the main genera in the large intestine were all anaerobes [16]. Also, the differences of luminal microbiota along the gastrointestinal tract in different animals, such as Bactrian camel, lamb, horse, and sheep, were studied [17-20]. However, the differences of MAM in different intestinal tract segments were rarely reported. In this study, the differences of LM and MAM in duodenum and in colon of mice were analyzed by 16S rRNA V3-V4 high-throughput sequencing, and the bacterial function and metabolic pathway prediction were conducted by PICRUSt (Phylogenetic investigation of the communities by reconstruction of the unobserved states).

## Materials and Methods

### Animals

Six SPF grade C57BL/6 male mice, aged 12 weeks and weighing 22-23g, were purchased from Vital River Laboratory Animal Technology Co. Ltd. (China). All mice were housed in an independent ventilator cage system under controlled conditions (humidity [55 ± 5%], light [12 h light/dark cycle], and temperature [24 ± 1°C]), and fed with standard sterilized rodent chow food and deionized water.

### Sample Collection

Two weeks later, the mice were sacrificed by deep anaesthetizing by isoflurane. The abdominal cavities were quickly opened, and the intestinal segments were removed. Then duodenum and colon were cut longitudinally separately, and the intestinal luminal contents were collected in sterile EP tubes respectively. After removing the contents, the remaining duodenum and colon were rinsed in precooled sterile phosphate buffer (PBS) twice to separate from mucus, loosely bound bacteria and digestive substances that adhered to the intestinal wall. Then, the intestinal mucosa was scraped from the inner intestinal surface using a lancet and collected in sterile EP tubes.

### Bacterial DNA Extraction

From feces and the scraped adherent-mucosa, bacterial DNA extracts were obtained by using the OMEGA DNA Kit (Omega Bio-Tek, USA) according to the manufacturer’s protocol. The quality and quantity of the DNA were evaluated by 1% (w/v) agarose gel electrophoresis in 0.5 mg/ml ethidium bromide and Nano Drop 2000 UV-vis spectrophotometer (Thermo Fisher Scientific, USA).

### High-throughput Sequencing and Bioinformatics Analysis

Genomic DNA was amplified with bacterial 16S rRNA gene (V3-V5 region)-specific primers: 338F (5’-ACTCCTACGGGAGGCAGC-3’) and 806R (5’-GG ACTACHVGGGTWTCTAAT-3’). The reverse primer contained a sample barcode, and both primers were connected with an Illumina sequencing adapter. PCR products were purified and the concentrations adjusted for sequencing on an Illumina Miseq PE300 system (Lianchuan Biotech Co., Ltd., China).

The raw reads of high-throughput sequencing were trimmed by removing their primers, barcodes, and adaptor sequences, and further screened and filtered as described by us [21]. Totally, 622,686 valid and trimmed sequences were obtained from all 24 samples, and average length was 440 bp per sequence. The total number of OTUs at 97% similarity level was 922. The minimum number of reads (17,658 reads) sub-sample was taken from each sample for subsequent analysis. The alpha diversity analysis was performed using Mothur software package (http://www.mothur.org/wiki/Main_Page ). A cluster dendrogram was constructed based on Bray-Curtis similarities. The principal co ordinates analysis (PCoA) and Analysis of similarities (ANOSIM) based on the Bray-Curtis distance were performed. To assess the effect size of each differentially abundant taxon, a metagenomic biomarker discovery approach was employed with LEfSe (linear discriminant analysis [LDA] coupled with effect size measurement, http://huttenhower.sph.harvard.edu/galaxy/) in which a nonparametric Wilcoxon sum-rank test was performed followed by LDA analysis. The bacteria network was generated using the CoNet plugin (version 1.0b7) for Cytoscape (version 3.7.1) on the basis of the nonparametric Spearman correlation coefﬁcients, with a minimal cutoff threshold of 0.6 (*p* < 0.01, Bonferroni corrected). We present correlation data for dominant genera (relative abundance, > 1%) that were detected in microbiota. Functional composition profiles of the gut microbiota were predicted from the 16S rDNA gene sequences using PICRUSt (Phylogenetic Investigation of Communities by Reconstruction of Unobserved States) with COG (Clusters of Orthologous Groups) and KEGG (Kyoto Encyclopedia of Genes and Genomes) database pathways. Significant differences were performed using Statistical Package for the Social Sciences (SPSS 19.0, USA). Results were expressed as means ± SEM (*i.e.* Standard Error of Mean) in individual experiment. Statistical tests were two-sided and a *p* < 0.05 was considered significant.

The raw data were deposited in Sequence Read Archive (SRA). The access number is PRJNA506118.

### Ethics Statement

This study was carried out in accordance with the recommendations of the Institute of Animal Care and Use Committee of Xinxiang Medical University, China. The experimental protocol for animal studies was reviewed and approved by the Ethics Committee of Xinxiang Medical University, China.

## Results

### Analysis of Bacterial Diversity and Evenness

According to the minimum number of sample sequences, 17,658 sequences were selected from each sample for analysis, and 922 OTUs with 97% similarity level were obtained. As shown in Table 1, no significant difference was observed in bacterial diversity and evenness between luminal content and mucosa in colon. However, the Shannon, Chao and Simpson indexes in duodenal luminal microbiota (D_LM) were significantly lower than these in duodenal mucosal microbiota (D_MAM), respectively (*p* < 0.05). In addition, the Shannon and Chao indexes in duodenum were significantly lower than these in colon, regardless of mucosa or luminal content (p <0.05).

### Clustering of the Bacterial Community 

**Fig. 1:**
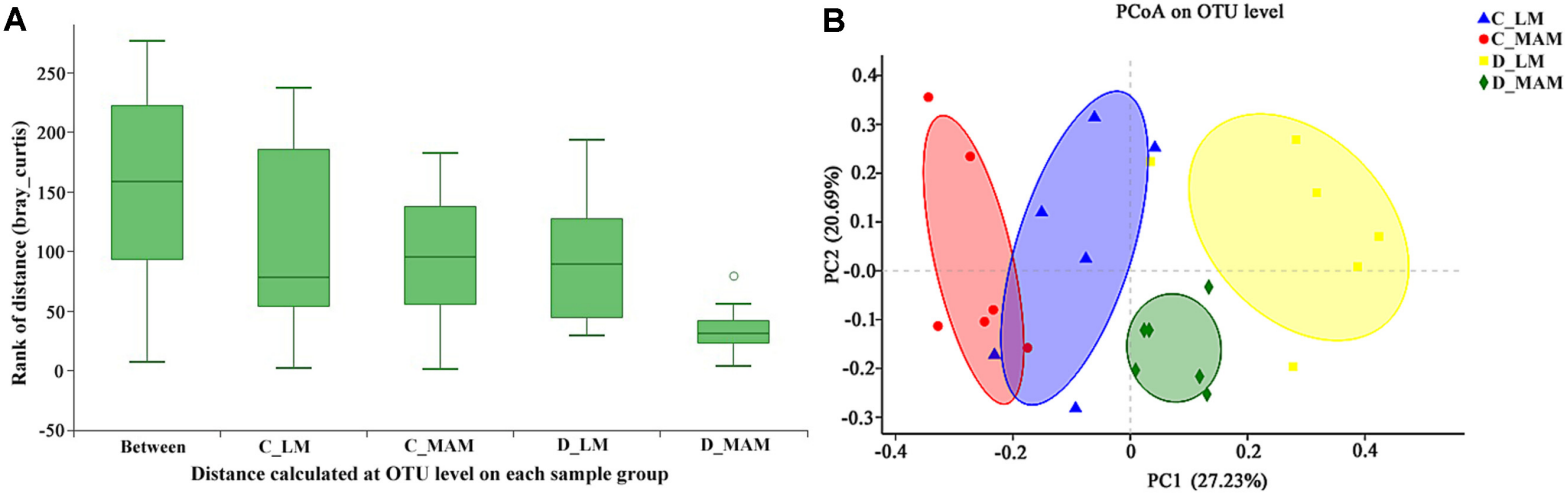
Comparative analysis between luminal and mucosal microbiota. (**A**) Analysis of similarities (ANOSIM) at OTU level was conducted. The middle line in the group “between” was higher than that in others; the differences in inter-treatment were bigger than those in intra-treatment. “0” meant the normalization point. (**B**) PC1 and PC2 explain 27.23% and 20.69% of variation, respectively. The statistics were performed based on Bray-Curtis distance, *n* = 6. C_LM: colonic luminal microbiota; C_MAM: colonic mucosal microbiota; D_LM: duodenal luminal microbiota; D_MAM: duodenal mucosal microbiota.

Analysis of similarities (ANOSIM) at OTU level was conducted (Fig. 1A). The R value was 0.5237, while *p* value was 0.001. The difference in microbiota inter-treatment was bigger than that in intra-treatment, indicating that the grouping was significantly meaningful. Principal co ordinates analysis (PCoA) based on bray_curtis showed that colonic luminal microbiota (C_LM) and colonic mucosal microbiota (C_MAM) were clustered closely with some overlap, while D_LM and D_MAM were separated completely (Fig. 1B).

### Changes in Bacterial Community Composition 

**Fig. 2:**
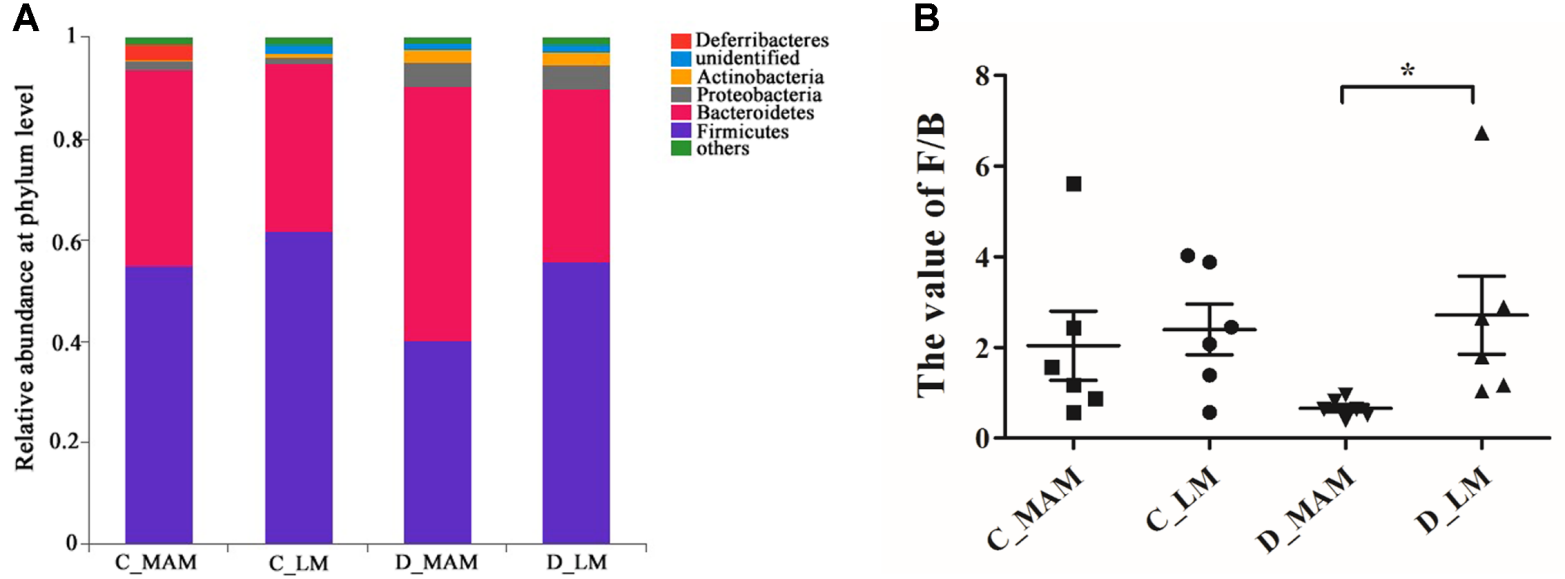
Community compositions of luminal and mucosal microbiota at phylum level. (**A**) Relative abundances of the main phyla. (**B**) The ratio of Firmicutes to Bacteroidetes (F/B). *indicated the significant differences (*p* < 0.05). The results are presented as the mean ± SEM; *n* = 6. C_LM: colonic luminal microbiota; C_MAM: colonic mucosal microbiota; D_LM: duodenal luminal microbiota; D_MAM: duodenal mucosal microbiota.

Based on the taxonomic results, 6 of all the detected 17 phyla exceeded 1%, and Firmicutes and Bacteroidetes were the most dominant (Fig. 2A). The percentage of Firmicutes was 61.76 ± 14.46% in C_LM, 55.20 ± 16.08% in C_MAM, 55.65 ± 20.88% in D_LM and 40.39% ± 9.16% in D_MAM. Bacteroidetes accounted for 33.22-50.14% among four groups. Due to the high inter-individual variabilities, no significant difference in Firmicutes or Bacteroidetes was observed between C_LM and C_MAM or between D_LM and D_MAM. However, the ratio of Firmicutes to Bacteroidetes (F/B) in D_LM was significantly higher than that in D_MAM (p <0.05). Besides, Deferribacteres in C_MAM was significantly more enriched than that in C_LM (2.93 ± 0.092% *vs.* 0.06 ± 0.038%) (*p* < 0.05) (Table S1 and S2).

**Fig. 3:**
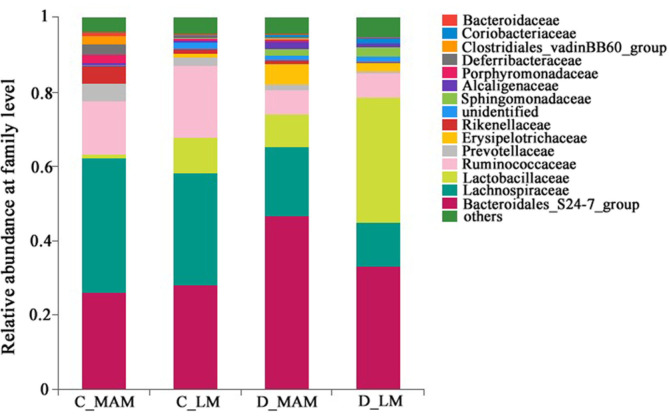
Community compositions of luminal and mucosal microbiota at family level. *n* = 6. C_LM: colonic luminal microbiota; C_MAM: colonic mucosal microbiota; D_LM: duodenal luminal microbiota; D_MAM: duodenal mucosal microbiota.

At the Family level, 29 families exceeding 1% were presented in Fig. 3, and *Bacteroidales*_S24-7, *Lachnospiraceae*, *Lactobacillaceae*, *Ruminococcaceae* were the most predominant families and accounted for 77.71%-87.28% in total. The comparison of luminal microbiota and mucosal microbiota in colon found that *Lactobacillaceae* (9.63 ± 7.69% *vs.* 0.84 ± 0.39%), *Erysipelotrichaceae* (0.83 ± 0.47% *vs.* 0.21± 0.12%), *Coriobacteriaceae* (0.34 ± 0.28% *vs.* 0.092 ± 0.039%) and unclassified *Mollicutes*_RF9 (0.019 ± 0.11% *vs.* 0.08 ± 0.06%) were enriched significantly more in C_LM than those C_MAM, while the percentage of *Rikenellaceae* (1.42 ± 0.55%*vs.* 4.70 ± 1.79%), *Deferribacteraceae* (0.060 ± 0.0085% *vs.* 2.93± 1.93%), *Clostridiales*_vadinBB60_group (0.27 ± 0.18% *vs.* 2.13 ± 0.89%) and unclassified *Gastranaerophilales* group (0.16 ± 0.12% *vs.* 0.80 ± 0.26%) in C_LM were significantly lower than these in C_MAM (*p* < 0.05). In duodenum, *Lactobacillaceae* in D_LM was obviously more enriched than that in D_MAM (33.65 ± 12.11% *vs.* 8.75 ± 3.72%, *p* < 0.05), while *Porphyromonadaceae* (0.089 ± 0.041% *vs.* 0.61 ± 0.44%) and Desulfovibrionaceae (0.072 ± 0.066% *vs.* 0.39 ± 0.11%) in D_LM were significantly decreased than these in D_MAM, respectively (*p* <0.05) (Table S1 and S2).

**Fig. 4:**
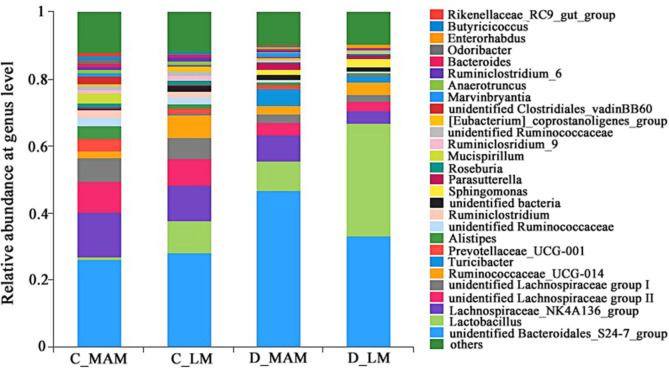
Community compositions of luminal and mucosal microbiota at genus level. *n* = 6. C_LM: colonic luminal microbiota; C_MAM: colonic mucosal microbiota; D_LM: duodenal luminal microbiota; D_MAM: duodenal mucosal microbiota.

At the genus level, 28 genera exceeding 1% were presented in Fig. 4, and unclassified *Bacteroidales*_S24-7, *Lactobacillus*, *Lachnospiraceae*_NK4A136, unclassified *Lachnospiraceae* group and *Ruminococcaceae*_UCG-014 were the most predominant genera and accounted for 58.52%-79.05% in total. In colon, compared with C_MAM, the abundances of the following were significantly increased in C_LM: *Lactobacillus*, unclassified_*Erysipelotrichaceae*, unclassified *Mollicutes*_RF9, *Erysipelatoclostridium*, *Enterorhabdus*, Family_ XIII_AD3011 group, Massilia and Parvibacter, while the following genera were significantly decreased: *Alistipes*, *Mucispirillum*, unclassified f__*Clostridiales*_vadinBB60_group, *Odoribacter*, *Marvinbryantia*, *Lachnoclostridium*, *Rikenellaceae*_ RC9_gut_group, unclassified *Gastranaerophilales* and [Eubacterium]_brachy_group (*p* < 0.05). In duodenum, just *Lactobacillus* was enriched significantly more in D_LM than that in D_MAM, while 10 genera (*Marvinbryantia*, *Ruminiclostridium*, Bilophila, *Odoribacter*, *Parabacteroides*, *Ruminococcaceae*_UCG-009, *Desulfovibrio*, *Lachnospiraceae*_FCS020_group, *Butyricimonas* and *Ruminococcaceae*_UCG-007) showed significantly lower abundances in D_LM than these in D_MAM (*p* < 0.05)(Table S1 and S2).

**Fig. 5:**
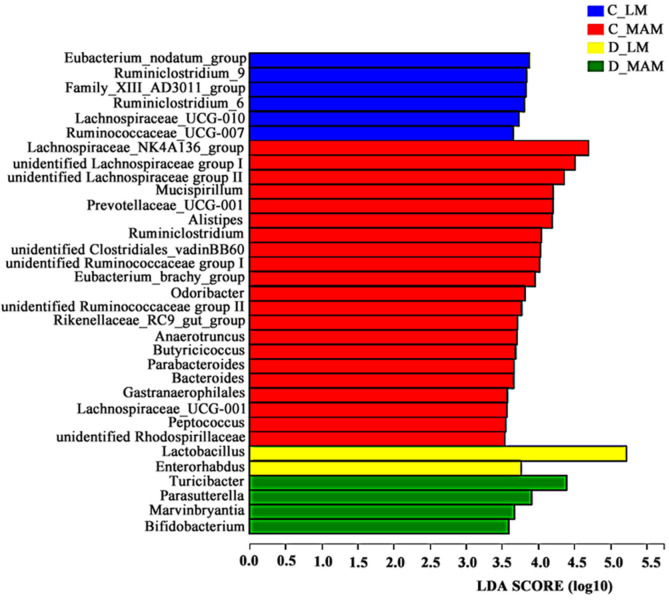
Linear discriminant analysis (LDA) coupled with effect size measurement (LEfSe) analysis of gut microbes in luminal and mucosal microbiota (LDA = 3.5). Non-parametric factorial Kruskal–Wallis (KW) sum-rank test was used. *n* = 6. C_LM: colonic luminal microbiota; C_MAM: colonic mucosal microbiota; D_LM: duodenal luminal microbiota; D_MAM: duodenal mucosal microbiota.

Linear discriminant analysis (LDA) coupled with effect size measurements was performed to detect the cladogram representation and the characteristic bacteria in luminal and mucosal microbiota from different intestinal positions. As shown in Fig. 5 (LDA = 3.5), the characteristic bacteria at genus level in C_LM were *Ruminiclostridium*_6, *Ruminiclostridium*_9, *Eubacterium*_nodatum group, *Ruminococcaceae*_UCG_007 and *Lachnospiraceae*_UCG_010, while in C_MAM they were *Lachnospiraceae*_NK4A136, *Mucispirillum*, *Alistipes*, *Ruminiclostridium*, *Odoribacter*, and so on. In duodenum, fewer characteristic genera were observed. In D_LM, *Lactobacillu* and *Enterorhabdus* were characteristic, while *Turicibacter*, *Parasutterella*, *Marvinbryantia* and *Bifidobacterium* were in D_MAM.

### Network Analysis of the Gut Microbiota at the Genus Level

To study the possible interactions among gut bacteria, 25 (in colon) and 20 (in duodenum) most dominant genera (relative abundance > 2%) were used to generate networks. The network included 24 nodes and 74 edges in colonic microbiota (Fig. 7A), and 17 nodes and 29 edges in duodenal microbiota (Fig. 7B). The clustering coefficient was 0.540 in colon and 0.516 in duodenum, respectively. In both networks, most of the network generated showed co-occurrent interactions (green lines), and the others exhibited mutual exclusions. The key genera of microbiota in colonic microbiota were unidentified *Bacteroidales*_S24-7-group (11 edges), *Roseburia* (10 edges) and *Ruminiclostridium* (10 edges), while those in duodenal microbiota were unidentified *Lachnospiraceae* group II (5 edges), *Lachnospiraceae*_NK4A136 group (5 edges), and *Stenotrophomonas* (5 edges). Interestingly, more interactions were observed among genera in colon than in duodenum. The average number of neighbors was 6.617 in colon and 3.412 in duodenum, respectively. The most abundant genus, unidentified *Bacteroidales* S24-7 group, was negatively related to *Lactobacillus* and was positively related to *Parasutterella* in duodenum, while it was negatively related to 8 genera and was positively related to 3 genera in colon. As one of the most abundant genera, *Lactobacillus* was just negatively related to 1 genus in duodenum and was negatively related to 5 genera in colon (Fig. 7).

### Microbiota Function Prediction

**Fig. 6:**
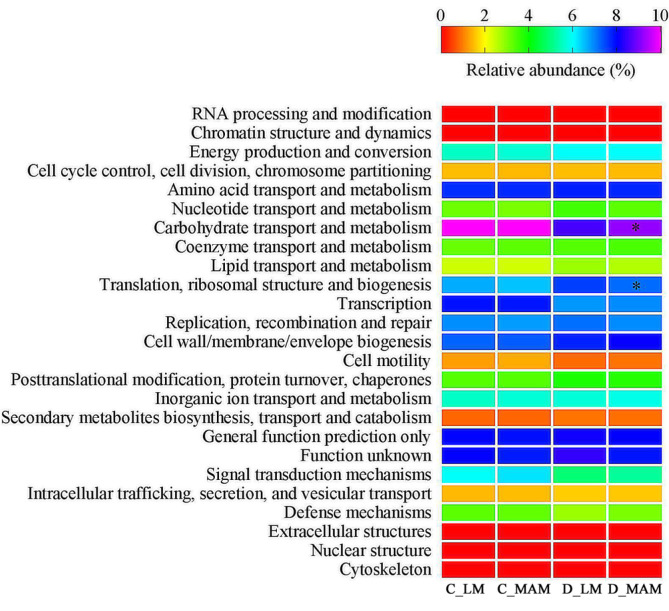
Predicted microbial function comparison based on COG. C_LM: colonic luminal microbiota; C_MAM: colonic mucosal microbiota; D_LM: duodenal luminal microbiota; D_MAM: duodenal mucosal microbiota. Statistics were conducted by two-sided Welch’s t-test, *n* = 6. *Significant differences were observed between D_LM and DM (*p* < 0.05).

**Fig. 7:**
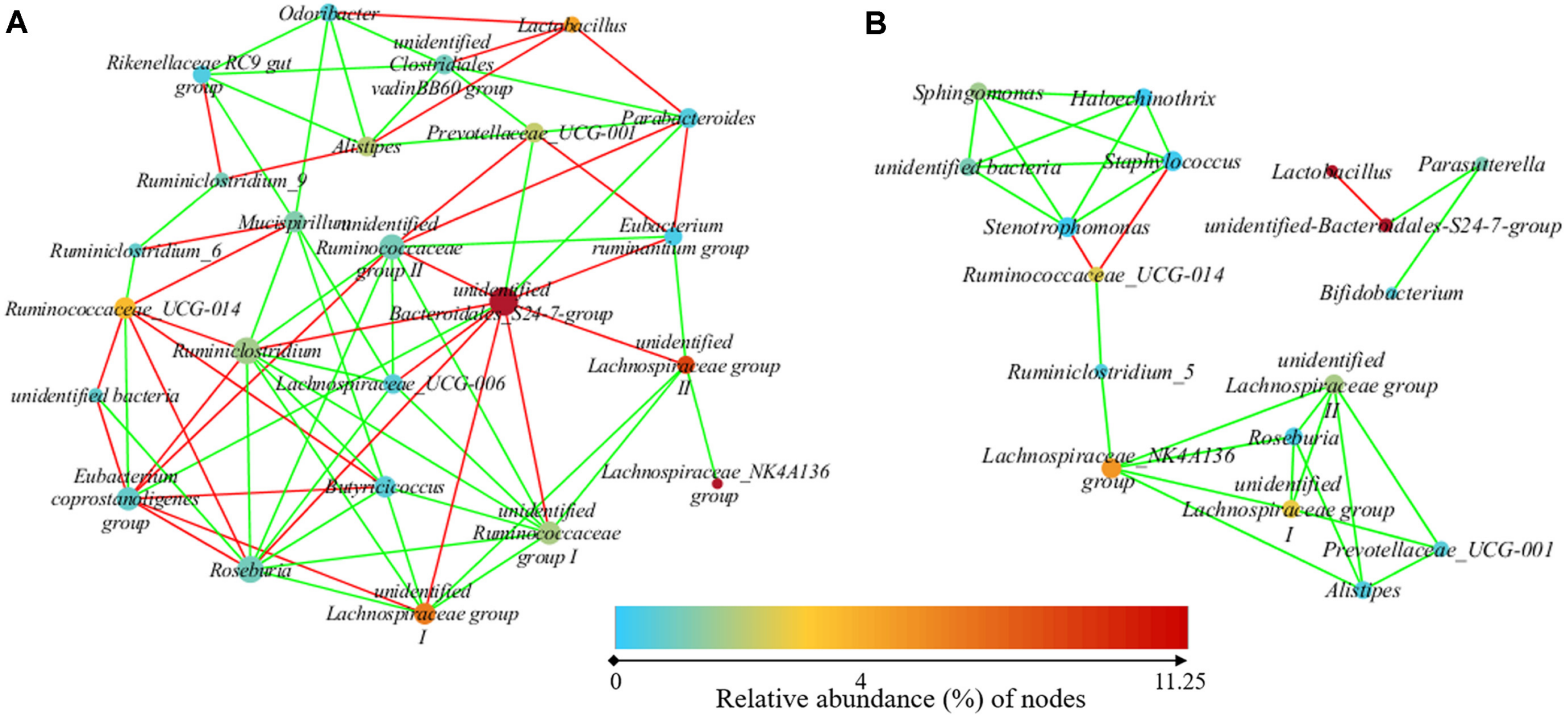
Network analysis of the gut microbiota at the genus level. (**A**) Network of the microbiota in colon; (**B**) Network of the microbiota in duodenum. The nonparametric Spearman correlation coefficients with a minimal cutoff threshold of 0.6 (*p* < 0.01, Bonferroni corrected) was used. Each node represents a bacterial genus, and the color of the node is proportional to the abundance. The size of the node is the degree of correlation with other genera. An edge indicates significant co-occurrence (green) and co-exclusion (red). *n* = 6. C_LM: colonic luminal microbiota; C_MAM: colonic mucosal microbiota; D_LM: duodenal luminal microbiota; D_MAM: duodenal mucosal microbiota

PICRUSt software was used to predict microbiota function. The COG (clusters of orthologous groups) classification evaluated the completeness of the transcriptome library and the phylogenetic annotation of newly sequenced genomes. The prediction results based on COG database showed that 24 gene functions were affected by different niches. No significant difference in the function genes was observed between C_LM and C_MAM. Compared with D_LM, “carbohydrate transport and metabolism” was significantly increased, while “translation, ribosomal structure and biogenesis” was decreased significantly in D_MAM (Fig. 6, *p* < 0.05).

The prediction of metabolic pathways based on KEGG showed that the “Metabolism” was the most dominant in all of 7 categories at level 1. Further analysis based on KEGG level 2 showed that 41 gene families were detected. Compared with C_LM, the proportion of “lipid metabolism” significantly declined in C_MAM (Fig. S1A, *p* < 0.05). Compared with D_LM, “endocrine system genes abundance” was increased while “carbohydrate metabolism” and “genetic information processing” were decreased in D_MAM (Fig. S1B, *p* < 0.05).

At KEGG level 3, 272 metabolic pathways were observed in total. In colon, the proportion of 3 metabolic predicted genes including “*Vibro cholera* pathogenic cycle,” “renal cell carcinoma” and “Parkinson’s disease” in C_MAM were significantly higher than these in C_LM, while the other 14 metabolic predicted genes obviously decreased, such as “glycolysis/gluconeogenesis,” “fructose and mannose metabolism,” “pyruvate metabolism” (Fig. S2, *p* < 0.05). In duodenum, compared with these in luminal content (D_LM), 12 predicted metabolic genes percentages in mucosal microbiota (D_MAM) were increased, such as “pyruvate metabolism,” “tyrosine metabolism,” “purine metabolism,” and “glycolysis/gluconeogenesis,” while the other 26 genes were decreased, such as “phenylalanin, tyrosine and trytophan biosynthesis,” “valine, leucine and isoleucine biosynthesis,” “ion-coupled transporters,” “trans-cription machinery,” and “phenylpropanoid biosynthesis” (Fig. S3, *p* < 0.05).

## Discussion

Consistent with previous studies [15, 22], both in luminal content and mucosa, higher bacterial diversity in colon than in duodenum were observed in this study. However, no significant difference of diversity and evenness was observed between colonic luminal microbiota with colonic mucosal microbiota, while higher diversity and evenness in mucosal microbiota (MAM) than in luminal microbiota (LM) were observed in duodenum. The microbiota is influenced by pH, intestinal motility, nutrient supplies and redox potential. The duodenum receives a steady stream of low pH digestions from the stomach. The duodenal mucosal microbiota may be relatively more stable than the content because of the cushion provided by intestinal mucosa and mucin. Thus, the diversity and evenness in duodenal mucosal microbiota were increased compared with that in duodenal luminal microbiota.

The dominant phyla in colon and duodenum were Firmicutes and Bacteroidetes. In this study, no significant difference was observed in the abundance of Firmicutes among four different intestinal positions, as was the case with Bacteroidetes. However, the Firmicutes/Bacteroidetes ratio in duodenal MAM showed smaller individual variation than in other three groups, and was significantly lower than that in duodenal LM. It has been reported that the F/B ratio of microbiota changes with various factors, such as diet, age, drug and disease [16, 21, 23, 24]. This study suggested that intestinal sampling location also affects the value of F/B ratio.

Distinct microbial populations within the mucosal and luminal niches in colon were reported [10, 11]. In this study, PCoA analysis exhibited the separation of LM and MAM in both colon and duodenum, and supported the variations in bacterial composition between lumen and mucosa. It was worth noting that duodenal LM and MAM clustered separately, whereas colonic LM and MAM clustered closer. Considering previous results of diversity and evenness, it can be concluded that the differences between LM and MAM in duodenum was greater than that between LM and MAM in colon.

It has been reported that the strictly anaerobic bacteria were enriched in the large intestinal samples and the facultative bacteria were enriched in the stomach and small intestine [14, 22]. This study also found that anaerobic *Ruminococcaceae* and *Lachnospiraceae* were the characteristic bacteria in colon, whereas *Lactobacillus* was enriched in duodenum. The difference of available oxygen in colon and in duodenum may be responsible for it.

Interestingly, the abundances of *Lactobacillus* in LM were significantly higher than that in MAM both in duodenum and in colon, and the percentage of *Lactobacillus* in duodenal LM as even over 30%. The important role of *Lactobacillus* in host digestion may explain its enrichment in LM both in duodenum and colon. *Lactobacillus* spp. participated in the degradation of lipids and simple sugars in the duodenum and jejunum and is also involved in the digestion of complex carbohydrates not digested by the host in the colon [25, 26]. An intense cross-talk occurs between the microbiota and the immunological niche in the intestinal mucosa, and the MAM may contribute more to interplay with the immune, metabolism and endocrine system [27]. Moreover, *Lactobacillus* species are able to survive in the presence of low pH, and some of them produce antimicrobial agents, allowing them to reduce the number of bacteria in the gut [26, 28]. It is notable that fewer interactions existed in duodenal microbiota than in colonic microbiota in this study. As one of the most abundant genera, *Lactobacillus* was interacted with 5 genera in colon, and with just 1 genus in duodenum. Maybe the low pH value in duodenum induced the enrichment of *Lactobacillus*, and was mainly responsible for the highest proportion of *Lactobacillus* and the lowest diversity of microbiota in duodenal LM. Considering more interactions with other genera, *Lactobacillus* might play a further important role in the microbiota by producing antimicrobial substances.

The mucosal microbiota played an important role in symbiotic relating with the hosting organism and in quorum sensing [29, 30], whereas luminal microbiota showed important digestion function [21-32]. In this study, metagenomic prediction at KEGG level 2 showed that duodenal MAM possessed stronger function than LM in “endocrine system,” while in “carbohydrate metabolism” duodenal LM was more active than duodenal MAM. Also, stronger “lipid metabolism” ability was predicted in colonic LM than in colonic MAM. It showed that LM focused on digestion while MAM contributed more in the interaction with host.

In conclusion, microbiota from intestinal luminal content and mucosa were different both in colon and in duodenum, and much more bacterial interactions existed in colon than those in duodenum.

## Supplemental Materials



Supplementary data for this paper are available on-line only at http://jmb.or.kr.

## Figures and Tables

**Table 1:** Bacterial diversity analyzed by high-throughput sequencing.

Indexes	C_LM	C_MAM	D_LM	D_MAM
Shannon	4.63 ± 0.26 a	4.69 ± 0.25 a	3.53 ± 0.51 c	4.20 ± 0.32 b
Chao	463.65 ± 54.98 ab	468.99 ± 39.52 a	330.89 ± 78.52 c	414.32 ± 42.15 b
Simpson	0.974 ± 0.009 a	0.978 ± 0.010 a	0.902 ± 0.036 b	0.963 ± 0.013 a
Coverage	0.996 ± 0.0005 a	0.996 ± 0.0009 a	0.997 ± 0.0009 a	0.996 ± 0.0013 a

Significant differences (*p* < 0.05) between groups are marked with the letters a, b, or c. The results are presented as mean ± SEM; *n* = 6. C_LM: colonic luminal microbiota; C_MAM: colonic mucosal microbiota; D_LM: duodenal luminal microbiota; D_MAM: duodenal mucosal microbiota.
