# Supplementary material for: The Differences between Luminal Microbiota and Mucosal Microbiota in Mice
Source: J Microbiol Biotechnol. 2019 Oct 22;30(2):287–95. doi: 10.4014/jmb.1908.08037 (PMC9728297; doi:10.4014/jmb.1908.08037)
Supplement: Supplementary file 1 [file JMB-30-2-287-supple.pdf]

J. Microbiol. Biotechnol.
https://doi.org/10.4014/jmb.1908.08037

jmb

Table S1. Relative abundances of significant changed bacteria at different levels (phylum, family and genus) in colon

Taxonomic group		C_LM (%)	C_MAM (%)	Variation
**Phylum**	Deferribacteres	0.06±0.04	2.93±1.20	↑*
**Family**	Lactobacillaceae	9.63±3.14	0.84±0.38	↓*
	Rikenellaceae	1.42±0.23	4.70±0.73	↑**
	Deferribacteraceae	0.06±0.04	2.93±1.20	↑*
	Clostridiales_vadinBB60_group	0.27±0.15	2.13±0.36	↑**
	Erysipelotrichaceae	0.83±0.19	0.21±0.05	↓*
	Unidentified Gastranaerophilales	0.16±0.05	0.80±0.26	↓*
	Coriobacteriaceae	0.34±0.11	0.09±0.02	↓**
	Mollicutes_RF9 group	0.19±0.05	0.08±0.03	↓*
**Genus**	Lactobacillus	9.63±3.14	0.84±0.38	↓*
	Alistipes	1.34±0.23	3.67±0.34	↑**
	Mucispirillum	0.06±0.04	2.93±1.20	↑*
	Unidentified Clostridiales_vadinBB60	0.27±0.15	2.13±0.36	↑**
	Odoribacter	0.22±0.04	1.24±0.47	↑*
	Marvinbryantia	0.38±0.14	0.99±0.14	↑*
	Lachnoclostridium	0.29±0.08	0.84±0.14	↑*
	Rikenellaceae_RC9_gut_group	0.08±0.03	1.03±0.43	↑**
	Unidentified Gastranaerophilales	0.16±0.05	0.80±0.26	↑*
	Unidentified Erysipelotrichaceae	0.26±0.07	0.03±0.00	↓**
	Unidentified Mollicutes_RF9	0.19±0.05	0.08±0.03	↓*
	Erysipelatoclostridium	0.21±0.06	0.03±0.01	↓**
	Enterorhabdus	0.17±0.04	0.05±0.01	↓*
	Family_XIII_AD3011_group	0.06±0.01	0.02±0.01	↓*
	[Eubacterium]_brachy_group	0.01±0.01	0.04±0.01	↑*
	Massilia	0.02±0.01	0	↓*
	Parvibacter	0.01±0.00	0	↓*

"↑" and "↓" represent the variation when the colonic mucosal microbiota (C_MAM) compared to the colonic luminal microbiota (C_LM).

n = 6 for each group. *P < 0.05, **P < 0.01.

Table S2. Relative abundance of significant changed bacteria at family and genus levels in duodenum

	Taxonomic group	D_LM (%)	D_MAM (%)	Variation
**Family**	Lactobacillaceae	33.65±4.94	8.75±1.52	↓**
	Porphyromonadaceae	0.09±0.06	0.61±0.18	↑*
	Desulfovibrionaceae	0.07±0.03	0.39±0.04	↑**
**Genus**	Lactobacillus	33.65±4.94	8.75±1.52	↓**
	Marvinbryantia	0.23±0.08	1.17±0.47	↑*
	Ruminiclostridium	0.15±0.11	0.36±0.10	↑*
	Bilophila	0.06±0.02	0.31±0.05	↑**
	Odoribacter	0.06±0.06	0.28±0.09	↑*
	Parabacteroides	0.02±0.01	0.27±0.09	↑*
	Ruminococcaceae_UCG-009	0.02±0.02	0.09±0.02	↑*
	Desulfovibrio	0.01±0.00	0.09±0.02	↑**
	Lachnospiraceae_FCS020_group	0.02±0.01	0.06±0.01	↑*
	Butyricimonas	0	0.06±0.04	↑*
	Ruminococcaceae_UCG-007	0	0.01±0.00	↑*

"↑" and "↓" represent the variation when the duodenal mucosal microbiota (D_MAM) compared to the duodenal luminal microbiota (D_LM).

n = 6 for each group. *$P < 0.05$, **$P < 0.01$.

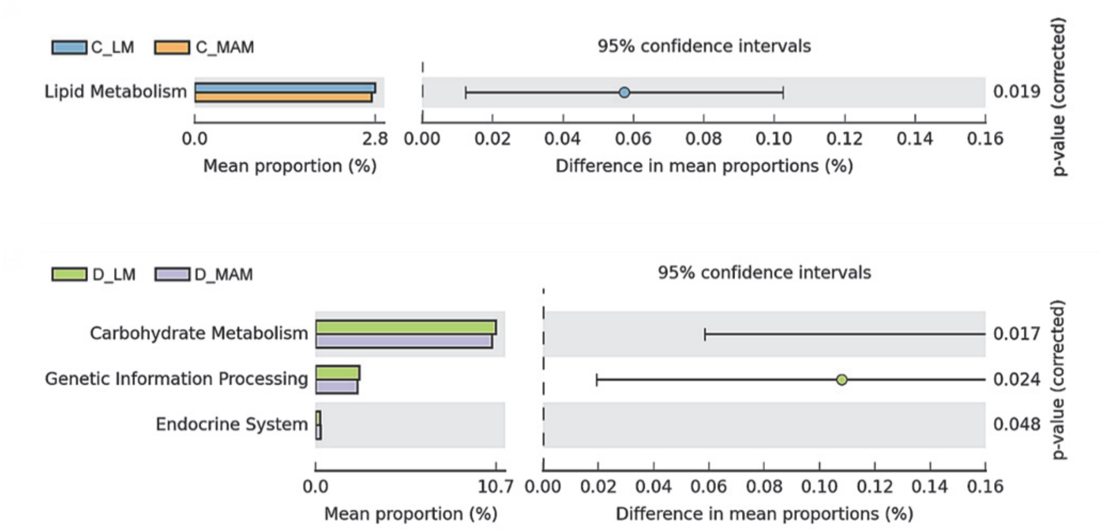

Fig. S1 Predicted microbial function comparison based on KEGG level-2. CC: colonic luminal content; CM: colonic mucosa; DC: duodenal luminal content; DM: duodenal mucosa. Statistics were conducted by two-sided Welch's t-test and Benjamini–Hochberg FDR correction, and the *P* values of different functions lower than 0.05 were shown.

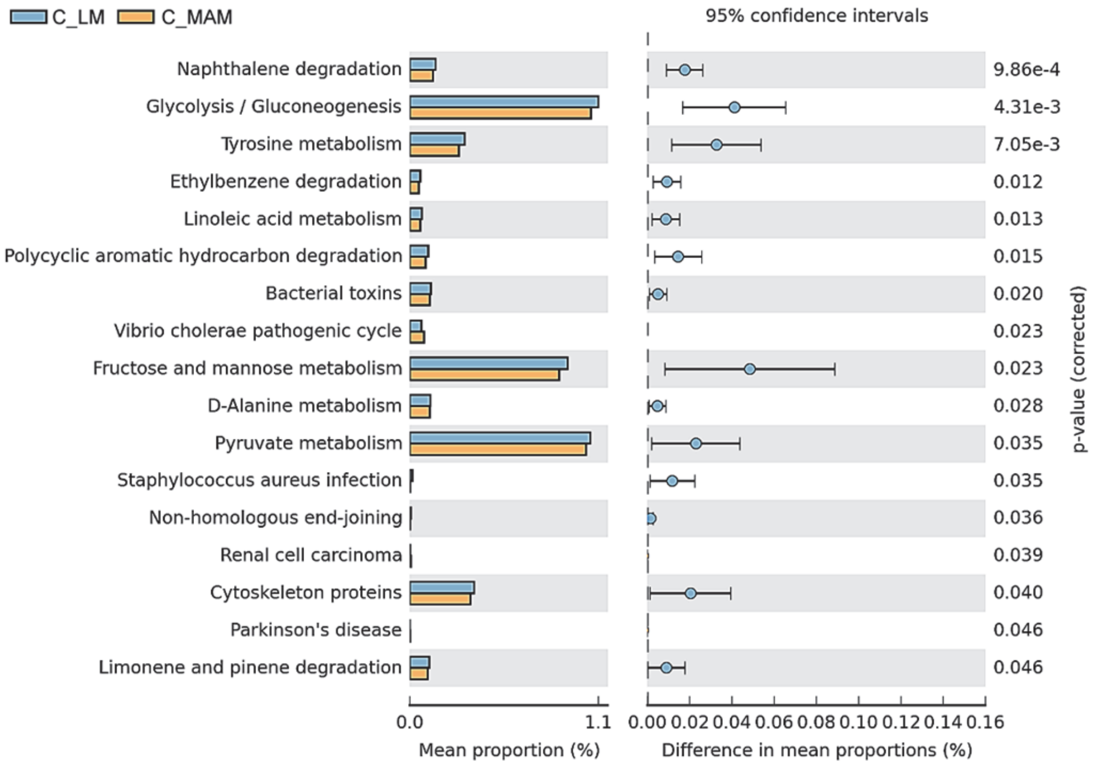

Fig. S2 Colonic predicted microbial function comparison based on KEGG level-3. CC: colonic luminal content; CM: colonic mucosa. Statistics were conducted by two-sided Welch's t-test and Benjamini–Hochberg FDR correction, and the *P* values of different functions lower than 0.05 were shown.

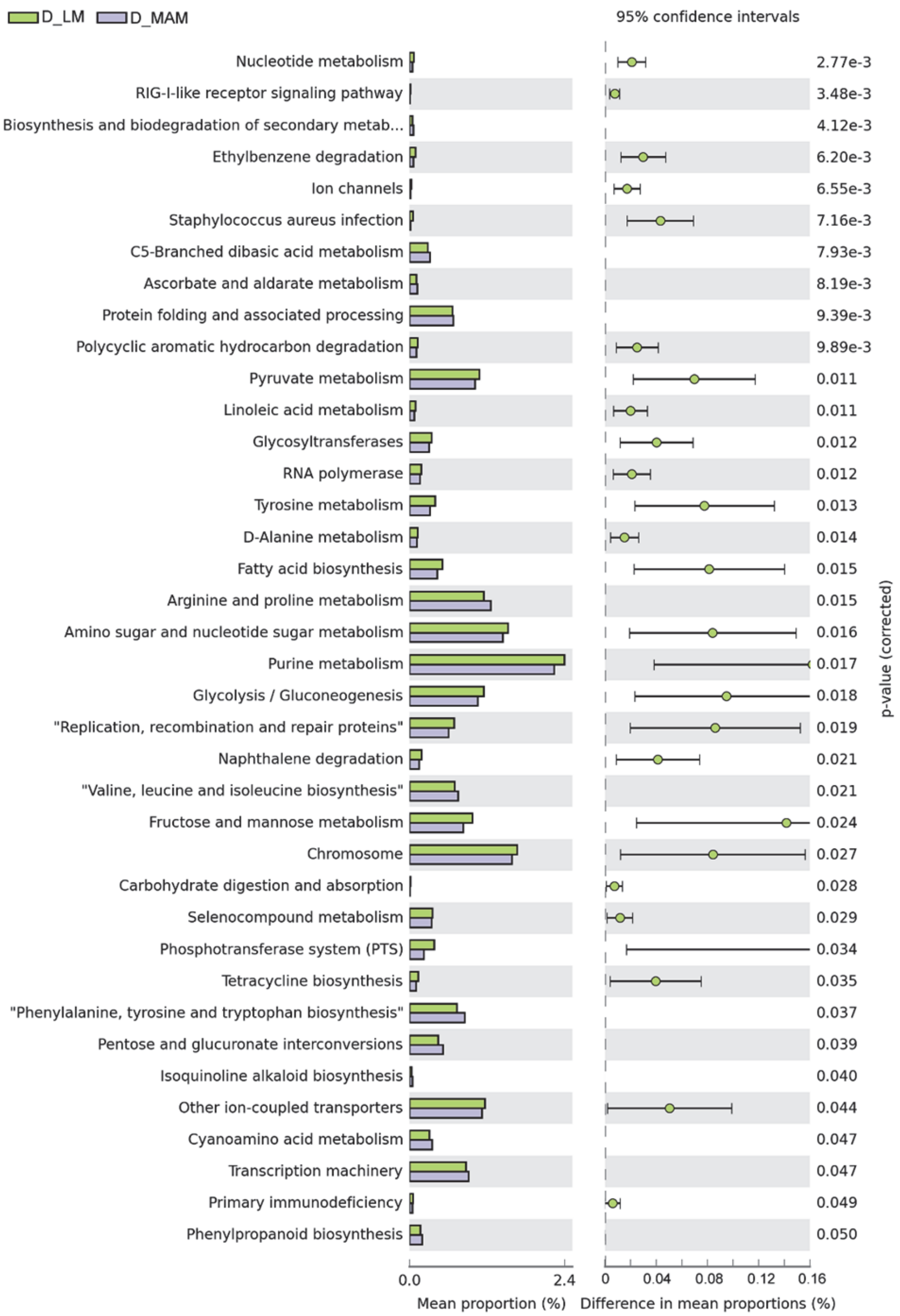

Fig. S3 Duodenal predicted microbial function comparison in based on KEGG level-3. DC: duodenal luminal content; DM: duodenal mucosa. Statistics were conducted by two-sided Welch's t-test and Benjamini–Hochberg FDR correction, and the *P* values of different functions lower than 0.05 were shown.

J. Microbiol. Biotechnol.
https://doi.org/10.4014/jmb.1908.08037

jmb

Table S1. Relative abundances of significant changed bacteria at different levels (phylum, family and genus) in colon

Taxonomic group		C_LM (%)	C_MAM (%)	Variation
**Phylum**	Deferribacteres	0.06±0.04	2.93±1.20	↑*
**Family**	Lactobacillaceae	9.63±3.14	0.84±0.38	↓*
	Rikenellaceae	1.42±0.23	4.70±0.73	↑**
	Deferribacteraceae	0.06±0.04	2.93±1.20	↑*
	Clostridiales_vadinBB60_group	0.27±0.15	2.13±0.36	↑**
	Erysipelotrichaceae	0.83±0.19	0.21±0.05	↓*
	Unidentified Gastranaerophilales	0.16±0.05	0.80±0.26	↓*
	Coriobacteriaceae	0.34±0.11	0.09±0.02	↓**
	Mollicutes_RF9 group	0.19±0.05	0.08±0.03	↓*
**Genus**	Lactobacillus	9.63±3.14	0.84±0.38	↓*
	Alistipes	1.34±0.23	3.67±0.34	↑**
	Mucispirillum	0.06±0.04	2.93±1.20	↑*
	Unidentified Clostridiales_vadinBB60	0.27±0.15	2.13±0.36	↑**
	Odoribacter	0.22±0.04	1.24±0.47	↑*
	Marvinbryantia	0.38±0.14	0.99±0.14	↑*
	Lachnoclostridium	0.29±0.08	0.84±0.14	↑*
	Rikenellaceae_RC9_gut_group	0.08±0.03	1.03±0.43	↑**
	Unidentified Gastranaerophilales	0.16±0.05	0.80±0.26	↑*
	Unidentified Erysipelotrichaceae	0.26±0.07	0.03±0.00	↓**
	Unidentified Mollicutes_RF9	0.19±0.05	0.08±0.03	↓*
	Erysipelatoclostridium	0.21±0.06	0.03±0.01	↓**
	Enterorhabdus	0.17±0.04	0.05±0.01	↓*
	Family_XIII_AD3011_group	0.06±0.01	0.02±0.01	↓*
	[Eubacterium]_brachy_group	0.01±0.01	0.04±0.01	↑*
	Massilia	0.02±0.01	0	↓*
	Parvibacter	0.01±0.00	0	↓*

"↑" and "↓" represent the variation when the colonic mucosal microbiota (C_MAM) compared to the colonic luminal microbiota (C_LM).

n = 6 for each group. *$P < 0.05$, **$P < 0.01$.
